# Physiotherapists' Experiences and Perceived Acceptability of Delivering a Knee Bracing Intervention for People With Symptomatic Knee Osteoarthritis in a Randomised Trial (PROP OA): A Qualitative Study

**DOI:** 10.1002/msc.70021

**Published:** 2024-12-18

**Authors:** Laurna Bullock, Melanie A. Holden, Clare Jinks, Evans Atiah Asamane, Dan Herron, Belinda Borrelli, Michael J. Callaghan, Fraser Birrell, Nicola Halliday, Michelle Marshall, Gail Sowden, Carol Ingram, John McBeth, Krysia Dziedzic, Nadine E. Foster, Sue Jowett, Sarah Lawton, Christian D. Mallen, George Peat

**Affiliations:** ^1^ School of Medicine Primary Care Centre Versus Arthritis Keele University Keele UK; ^2^ Centre for Musculoskeletal Research Keele University Keele UK; ^3^ Institute of Applied Health Research University of Birmingham Birmingham UK; ^4^ Department of Psychology School of Health Education Policing and Sciences Staffordshire University Stoke‐on‐Trent UK; ^5^ Henry M Goldman School of Dental Medicine Boston University Boston Massachusetts USA; ^6^ Manchester Centre for Health Psychology Division of Psychology and Mental Health School of Health Sciences Manchester Academic Health Science Centre The University of Manchester Manchester UK; ^7^ Centre for Musculoskeletal Research Manchester Academic Health Sciences Centre The University of Manchester Manchester UK; ^8^ Faculty of Health and Education Manchester Metropolitan University Manchester Manchester UK; ^9^ Manchester University Hospitals NHS Foundation Trust Manchester UK; ^10^ MRC‐Versus Arthritis Centre for Integrated Research Into Musculoskeletal Ageing Newcastle University Newcastle upon Tyne UK; ^11^ Northumbria Healthcare NHS Foundation Trust North Tyneside UK; ^12^ Keele Clinical Trials Unit Keele University Keele UK; ^13^ Connect Health Newcastle Upon Tyne UK; ^14^ Research User Group Primary Care Centre Versus Arthritis School of Medicine Keele University Keele UK; ^15^ School of Primary Care Population Sciences and Medical Education University of Southampton Southampton UK; ^16^ Impact Accelerator Unit Keele University Keele UK; ^17^ Surgical Treatment and Rehabilitation Service (STARS) Education and Research Alliance The University of Queensland and Metro North Health Brisbane Australia; ^18^ Health Economics Unit Department of Applied Health Research School of Health Sciences University of Birmingham Birmingham UK; ^19^ Centre for Applied Health & Social Care Research (CARe) Sheffield Hallam University Sheffield UK

**Keywords:** bracing, knee, osteoarthritis, physiotherapy, qualitative, randomised controlled trial

## Abstract

**Objectives:**

To explore physiotherapists' experiences and perceived acceptability of delivering a bracing intervention for knee osteoarthritis (OA) in the ‘PROvision of braces for Patients with knee OA’ (PROP OA) randomised controlled trial.

**Method:**

Semi‐structured telephone interviews with consenting physiotherapists who received the PROP OA training programme and delivered the knee bracing intervention (advice, information and exercise instruction plus knee brace matched to patients' clinical and radiographic presentation and with adherence support). Interviews were recorded and transcribed verbatim. Two‐stage analytic framework: inductive thematic analysis preceded mapping to constructs of the Theoretical Framework of Acceptability.

**Results:**

Eight physiotherapists were interviewed and six key themes were developed. ***Perceptions of the training programme*** were generally positive, but additional formal training and experiential learning consolidated confidence and skills in novel intervention components. ***Advice, information, and exercise instruction*** reflected usual physiotherapy care for knee OA. Physiotherapists were confident in ***delivering the knee brace***, but determining the pattern of knee OA to inform brace type selection was challenging. Physiotherapists valued ***brace adherence enhancing strategies*** and the ***follow‐up appointment*** to facilitate adherence. ***Perceived impact of the bracing intervention for people with OA*** was positive. The bracing intervention was perceived as acceptable, although improving self‐efficacy to deliver novel intervention components (e.g., reading x‐rays) would enhance acceptability.

**Conclusion:**

The complex knee bracing intervention was broadly perceived as acceptable by physiotherapists. If implemented within clinical practice beyond the trial, physiotherapists might benefit from not only initial training in brace selection but also ongoing support and mentoring to increase self‐efficacy in delivery.

## 1 Introduction

Symptomatic knee osteoarthritis (OA) affects approximately 365 million adults worldwide (Yang et al. 2023) and has a significant impact on population health, healthcare demand, and societal costs. Total direct and indirect costs associated with OA are currently estimated at 1%–2.5% of gross domestic product (GDP) in high‐income countries (Hiligsmann et al. 2013). The high burden of knee OA is predicted to rise even further with an ageing population and increasing levels of obesity and multimorbidity (Pabinger, Lothaller, and Geissler 2015). As there is no cure for OA, treatments that control symptoms and improve function are the focus of healthcare (Hunter and Bierma‐Zeinstra 2019).

Bracing is one of several non‐pharmacological treatment options for knee OA. However, due to limitations in the evidence base, including heterogeneous research findings and low‐quality evidence, current clinical guidelines offer contradictory and conflicting recommendations on their use (American Academy of Orthopaedic Surgeons 2021; Bannuru et al. 2019; Bichsel et al. 2022; Kolasinski et al. 2020; NICE 2022; Royal Australian College of General Practitioners 2018). Bracing for knee OA represents a class of complex interventions that comprise a variety of devices (including valgus/varus braces, patellofemoral braces, neutral stabilising, and soft sleeve braces) for different presentations of knee OA (including tibiofemoral, patellofemoral, and mixed knee OA) with several proposed mechanisms of action (including biomechanical, neuromuscular, and psychological) (M. Holden et al. 2023). Previous interventions include different components (e.g., brace fitting, encouraging brace adherence), target a range of behaviours (e.g., donning the brace, wearing the brace over time, using the brace within a broader self‐management programme), and require varying levels of skill and expertise to apply (M. Holden et al. 2023).

Motivational interviewing (MI) has the potential to support brace adherence as one component of a complex bracing intervention. MI is a well‐evidenced and person‐centred approach to build patients' intrinsic motivation and resolve ambivalence about behaviour change (e.g., adherence to brace use; Borrelli et al. 2007).

Physiotherapists are well placed to deliver complex knee brace interventions for people with knee OA. They are the largest group of allied health professionals who manage musculoskeletal problems within the UK National Health Service (NHS) and are already commonly involved in the treatment of people with knee OA through the provision of advice, information, and exercise. However, physiotherapists rarely provide knee brace interventions for knee OA within randomised controlled trials (RCTs), and knee braces are not commonly provided to people with knee OA in routine physiotherapy practice (Hagen et al. 2016; M. A. Holden et al. 2008). As such, the acceptability of physiotherapists delivering brace interventions for knee OA is currently unknown.

### 1.1 The PROP OA RCT

The ‘PROvision of braces for Patients with knee OA’ (PROP OA) RCT is a multicentre, pragmatic, two‐parallel group, single‐blind, superiority RCT of eligible participants with symptomatic knee OA (ISRCTN28555470). A full description of the RCT has been published elsewhere (M. A. Holden et al. 2021). The PROP OA RCT aims to determine the clinical and cost‐effectiveness of adding physiotherapy‐delivered knee bracing (matched to participants' clinical and radiographic presentation and with adherence support) to advice, written information and exercise instruction compared with advice, written information, and exercise instruction alone in adults with symptomatic knee OA. The first 9 months of PROP OA RCT recruitment served as an internal pilot phase during which we embedded a qualitative study to explore the experiences and perceived acceptability of the bracing intervention to physiotherapists involved in the trial.

## 2 Methods

### 2.1 Description of the Intervention

In summary, the intervention was delivered by UK Health and Care Professions Council (HCPC) registered physiotherapists who undertook the PROP OA training programme and included knee bracing (matched to participants' clinical and radiographic presentation and with adherence support) plus advice, written information and exercise instruction. The intervention was delivered via an initial 1‐h face‐to‐face treatment session, a 30‐min follow‐up appointment 2 weeks later, and automated motivational prompts to enhance brace adherence sent via SMS text message over 6 months (which physiotherapists are not involved with and hence are not the focus of this study). These components, and the associated PROP OA training programme, are collectively described as the ‘knee bracing intervention’ hereafter, further described below and in full elsewhere (M. A. Holden et al., 2021).

#### 2.1.1 Initial Treatment Session

Physiotherapists provided participants with verbal advice on the pathogenesis and prognosis of knee OA, benefits of exercise, physical activity and weight loss, simple self‐help advice on pain management; written information in the form of the OA guidebook (www.keele.health/osteoarthritis‐resources/#osteoguide) adapted for the PROP OA RCT by removal of information about bracing to reduce potential contamination; and instruction to complete a home‐based lower limb exercise programme focussing on muscle strengthening, knee range of movement and proprioception. Physiotherapists also provided either a patellofemoral (Bioskin Q Brace), tibiofemoral unloading (Össur Unloader One), or neutral stabilising knee brace (Össur Formfit Knee Hinged) according to whether participants' pattern of knee OA was predominantly patellofemoral, tibiofemoral (medial/lateral), or multi‐compartmental. The choice of brace was based on the predominant knee compartment affected (from clinical assessment and x‐rays (Appendix 1)), and with consideration of the patients' current and desired level of physical activity, ability to don/doff the brace, willingness to wear the brace type, and immediate symptom response. Physiotherapists were instructed to fit the brace to ensure maximum comfort (e.g., adjusting and cutting straps to match participants' body shape and size), to give advice about brace dose, and to provide verbal and written information on brace application and care, including cleaning instructions and what to do in instances of slippage, discomfort, or skin irritation. Physiotherapists employed brief MI techniques and gave participants a diary to monitor and record brace use, barriers to use, and potential solutions.

#### 2.1.2 Follow‐Up Treatment Session

The primary purpose of the follow‐up session was for the physiotherapists to check response to, and fit of, the brace. Advice on further brace use was provided as appropriate (e.g., participants reporting good tolerance and benefit would be advised to increase brace use). If the brace was not being tolerated, physiotherapists could change the brace type. Adherence to brace use was reviewed based on information provided by the participant within the brace diary and addressed using brief MI techniques.

#### 2.1.3 Physiotherapist Training Programme

All physiotherapists involved in the internal pilot attended a 3‐day face‐to‐face PROP OA training programme prior to the RCT starting and received a supporting manual. Training covered all aspects of the brace intervention, including the provision of advice, information and exercise; allocation and fitting of the knee brace (using clinical assessment, and reading and interpretation of plain knee x‐rays to judge compartmental involvement); provision of advice and information on brace dose and maintenance); and brief MI to facilitate motivation to adhere to brace use. Training was provided didactically and through interactive group discussions, problem solving, case examples and role play. Physiotherapists were offered regular virtual group ‘booster sessions’ with the MI trainer to discuss problems, challenges and successes. Videos produced by the trial team and brace manufacturers were also made available for physiotherapists to review, and braces were provided for physiotherapists to practice with.

### 2.2 Qualitative Study

#### 2.2.1 Participants

Physiotherapists participating in the PROP OA RCT at the time of the internal pilot were invited to take part in an interview. The interview was completed 3 months after the RCT commenced recruitment to ensure that physiotherapists had experience of delivering the bracing intervention.

#### 2.2.2 Data Collection

Following informed consent, one‐to‐one semi‐structured telephone interviews were undertaken with physiotherapists between May 2020 and January 2021. A topic guide (Appendix 2) was initially developed by members of the PROP OA study team (including MH, CJ and DH), informed by the Theoretical Framework of Acceptability (TFA) domains (Sekhon, Cartwright, and Francis 2017). The topic guide was iteratively updated using feedback from the wider multidisciplinary PROP OA study team, including public contributors, and iteratively refined during data collection and analysis. All interviews lasted for up to 1 hour and were conducted by an experienced post‐doctoral qualitative researcher who was not involved in delivering the physiotherapist training programme (EAA). Interviews were digitally audio‐recorded, transcribed, checked for accuracy, and anonymised for analysis.

#### 2.2.3 Analysis

A two‐stage analytic framework was used: firstly, inductive thematic analysis and secondly, themes were mapped to the TFA domains (affective attitude, burden, intervention coherence, perceived effectiveness, opportunity costs, self‐efficacy, ethicality, global acceptability). For analysis, each interview transcript was initially read and re‐read (familiarisation) by LB (an experienced post‐doctoral qualitative researcher) who identified and applied initial codes to discrete parts of the data that represented a particular concept using NVivo12. Data were closely examined for similarities and differences, leading to the development of a codebook, code refinement, and theme development. Themes were then mapped to TFA domains to explore meaning in the data and facilitate understanding of perceived acceptability of the brace intervention. Regular discussions with the experienced multidisciplinary team (including members with clinical physiotherapy and methodological expertise (MH, CJ)) throughout the analytical process facilitated interpretation, ensuring trustworthiness.

#### 2.2.4 Patient and Public Involvement

Public contributors with lived experience of OA attended dedicated workshops to inform the design of the PROP OA RCT, as described elsewhere (M. A. Holden et al., 2021). Qualitative study design, interview data collection and data interpretation were shaped by public contributors who attended trial management group meetings as core members of the PROP OA study team.

## 3 Results

### 3.1 Recruitment and Sample Characteristics

All nine physiotherapists who were participating in the PROP OA RCT at the time of the internal pilot were invited to take part in an interview. One physiotherapist who stopped contributing to the RCT declined, resulting in 8 physiotherapists being interviewed. As shown in Table 1, six physiotherapists (75%) had been qualified for 10 years or longer. Three physiotherapists had previous postgraduate training in OA (*n* = 3.38%) and only 1 (13%) had postgraduate training in braces for knee OA. Three physiotherapists (38%) self‐reported previous postgraduate training in MI, consisting of online training modules and other study training, with no formal assessments.

**TABLE 1:** Characteristics of participating physiotherapists.

Total *n*	8
Sex *n*	
Female	3
Band *n*	
5/6	5
7/8a	3
Years qualified mean (range)	16 (4–36)
Postgrad. training in OA *n*	
Yes	3
No	5
Postgrad. training in braces for knee *n*	
Yes	1
No	7
Postgrad. training in MI *n*	
Yes	3
No	4
Unknown	1

*Note:* Characteristics self‐reported by physiotherapists at the PROP OA training session.

Abbreviations: MI, Motivational Interviewing; OA, osteoarthritis.

### 3.2 Key Themes Overview

Six key themes were developed: (1) perceptions of the training programme; (2) advice, information, and exercise instruction; (3) delivering the knee brace (with subthemes of acceptability of knee braces, decisional certainty in selecting brace type, fitting the knee brace), (4) adherence enhancing strategies (with subthemes of MI and the knee brace diary); (5) the follow‐up appointment; and (6) perceived impact of the bracing intervention. A summary of themes and subthemes is provided below, with illustrative quotes provided. Table 2 maps the themes to the TFA.

**TABLE 2:** Overview of qualitative findings mapped to the theoretical framework of acceptability (TFA).

TFA construct	Physiotherapist findings	Theme
Affective attitude (feelings and emotions related to PROP OA)	‐ Training on the whole sufficiently met physiotherapists' needs.	1
	‐ X‐rays deemed useful and interesting.	3
	‐ Positive language used to describe the knee bracing intervention.	3
	‐ Motivational interviewing deemed interesting and enjoyable.	3
	‐ Participants viewed as having a positive perception of the brace.	6
Burden (effort required)	‐ Overall, the knee bracing intervention was deliverable, but did involve some burden, including lack of familiarity, learning new skills, and time for delivery.	3
Intervention coherence (the purpose of PROP OA and how it works)	‐ Physiotherapists generally followed the bracing intervention protocol showing understanding.	3
	‐ Good understanding of the function of the follow‐up appointment.	5
	‐ Physiotherapists provided thorough explanations.	2
Perceived effectiveness (effectiveness of interventions)	‐ Participants were generally deemed to gain benefits from brace use.	6
	‐ Diaries were seen as a mechanism to support discussions and prompt brace use.	4
	‐ Motivational interviewing perceived as a powerful tool.	4
	‐ Advice, information and exercise instruction perceived as usual care in line with best practice guidance.	2
Opportunity cost (giving up values or benefits)	‐ Acknowledged that wearing the brace could be beneficial but could also result in adverse reactions.	6
Self‐efficacy (confidence)	‐ On the whole physiotherapists were confident in delivering the knee bracing intervention.	3
	‐ Least confident in x‐ray reading and motivational interviewing. Formal training and informal practise used to increase this self‐confidence.	1, 3
Ethicality (fit with values)	‐ To be fair for future implementation, the knee bracing intervention should be offered as part of usual NHS care to those likely to benefit the most, and people should be able to accept or decline the intervention.	6
Global acceptability (appropriateness based on anticipated/experiential cognitive and emotional responses)	‐ Largely positive reflections on the knee bracing intervention overall.	1, 2, 3, 4, 5, 6

*Note:* Themes: 1) perceptions of the training programme; 2) advice, information, and exercise instruction; 3) delivering the knee brace (with subthemes of acceptability of knee braces, decisional certainty in selecting brace type, fitting the knee brace); 4) adherence enhancing strategies (with subthemes of MI and the knee brace diary); 5) the follow‐up appointment; 6) perceived impact of the bracing intervention.

### 3.3 Perceptions of the Training Programme

Many physiotherapists thought that the training was useful and relevant to prepare them for participating in the PROP OA RCT. Whilst some physiotherapists specifically highlighted that training on reading knee x‐rays to determine compartmental involvement and brief MI had worked well and fulfiled their training needs, others continued to lack confidence in these areas.

*I think that’s [reading and interpreting x‐rays to determine compartmental involvement] probably the least bit I’m confident with, but apart from that all of the parts of the training have been really useful.* (P01)

In addition to the formal PROP OA training provided, physiotherapists also reflected upon additional self‐initiated training that they undertook. One physiotherapist attended a formal course on MI. This decision was motivated by their interest in MI after attending the PROP OA training but with a perception that they had unmet training needs in this area. The physiotherapist reflected that this extra training would support trial delivery but would also be transferable to their broader role.

*I took it on my own back to go on a day’s course of MI (…) because I felt I hadn’t been trained up enough or adequately enough in that area. So, I’ve done additional training in that area just for myself. Which is useful in my day job as well, it’s not just for PROP OA.* (P08)

Many physiotherapists noted the importance of informally practising skills introduced in the PROP OA training to increase confidence when delivering the bracing intervention. They described practising fitting braces on themselves, colleagues, and family members, guided by video resources provided as part of the PROP OA training, to facilitate ‘hands‐on’ experiential learning.

*I got together with a couple of colleagues to have a bit of a practise to put on some of the different braces*. (P02)

Physiotherapists provided recommendations to improve the PROP OA training. This included more time to practise brief MI, brace fitting and reading/interpreting compartmental involvement on the x‐rays. Physiotherapists also recommended that time spent on the background and theoretical information could be reduced.

*motivational interviewing was different, that was tricky. I could’ve spent longer I think actually having some training on motivational interviewing.* (P08)

*I think maybe having a little bit longer on the x‐rays may have been useful at times*. (P04)

The bracing intervention required physiotherapists to implement multiple intervention components alongside trial processes. Continued practice following the PROP OA training was perceived as important to facilitate intervention integration into clinical practice.

*I used the analogy of driving a car. When you first drive a car, you’ve got lots of things to think about. Because physios aren’t erm – they’re not used to using braces and motivational interviewing isn’t something that’s completely commonplace, so putting the two things together does require a bit of practise*. (P02)

*I might have to be concentrating so hard on putting the brace on that some of the change talk is kind of in the background because of that so I think that, I would hope, is something that would improve with practise*. (P04)

### 3.4 Advice, Information, and Exercise Instruction

It was noted by some physiotherapists that the content of advice, information and exercise instruction reflected best practice guidance, and most physiotherapists reported feeling confident in its delivery. This was linked to it being perceived as a core component of standard physiotherapy treatment provision.

*It’s bread and butter, it’s what we would do if we had a patient with OA knee.* (P08)

Some physiotherapists, however, conceptualised the advice, information and exercise instruction expected in PROP OA as different from their usual practice.

*I think, generally speaking, it’s the kind of practise of talking through best practice [advice, information, exercise] to a patient in the way that the study team want*. (P04)

The OA guidebook was perceived to be an appropriate and useful resource in supporting the delivery of advice and information.

*The information booklet that you’ve got is actually really useful to have those conversations for the patients because it’s well written, it’s written in combination with patients, so the wording is correctly pitched*. (P01)

However, some challenges to the delivery of advice, education and exercise instruction were noted, including lack of engagement by some trial participants, and describing OA in an understandable way.

*as many times as you try and describe what arthritis is to patients, it can be very difficult because you’ve got this kind of constant… of trying not to use too many medical terminologies, so trying to speak in kind of layman’s terms whilst trying to sum up and summarise kind of sort of medical terminology can be quite difficult*. (P03)

### 3.5 Delivering the Knee Brace Intervention

#### 3.5.1 Acceptability of Knee Braces

Overall, physiotherapists were accepting braces as a potentially helpful treatment for knee OA. The opportunity to provide a brace increased the physiotherapists' satisfaction with the care provided.

*Some patients report an instantaneous feeling of satisfaction from having received the brace(…) If you give them [patients] the brace it definitely is a much more pleasing and satisfying thing to do, I must admit.* (P06)

#### 3.5.2 Decisional Certainty in Selecting Brace Type

Physiotherapists reflected on selecting a brace type according to the participants' pattern of knee OA, a decision which was predominantly based on clinical assessment and x‐ray findings. Most physiotherapists felt very confident in undertaking the clinical assessment, describing it as their “bread and butter”. However, some described the challenge of using information gathered from the clinical assessment and x‐ray findings to determine the pattern of knee OA, particularly when more than one compartment (medial/lateral tibiofemoral, patellofemoral) was involved. Confidence in reading and interpreting x‐rays for compartmental involvement varied across the physiotherapists, which largely depended on their experience.

*I’m probably still least confident in making decisions on the x‐rays, but that’s probably because I wouldn’t have made a clinical judgement on an x‐ray before.* (P01)

Almost all physiotherapists reflected upon the discrepancy that could occur between clinical examination and x‐ray findings, and how at times this may have added complexity to or impeded their clinical judgement.

*It’s a bit of a grey area at times, it’s not clear cut (…) their symptoms don’t always marry up with what the x‐ray’s showing*. (P08)

When there was a discrepancy, physiotherapists reportedly relied on the findings of their clinical assessment to select a brace type.

*as long as you’ve got a fairly thorough subjective assessment, finding out what they’re aggravating and easing factors are, erm, that should then help you pick the right brace*. (P03)

#### 3.5.3 Fitting the Knee Brace

Whilst some physiotherapists reported no challenges in fitting knee braces, others found it difficult due to it being a new skill.

*I don’t prescribe braces in my clinical practice so it’s all new (…) all in all that is the most technically challenging aspect of the study, is getting the brace on, getting it fitted well, getting people trialing it and then working out how they are going to engage with it as a kind of new treatment.* (P04)

Physiotherapists felt that their confidence when fitting the brace was important to increase the participant's motivation to adhere to the treatment.

*The practise that you put in to make sure that you’re confident with the adjustments you need to make with the brace then pays off with compliance for the patient. Because actually they are then more confident it’s going to work for them and therefore they’re more willing to try*. (P01)

The Össur off‐loader brace was deemed most challenging to fit by many physiotherapists because it had more components to consider than other braces.

*I find [the Össur unloader brace] a hard brace to use, I find it erm a nightmare to measure and apply*. (P08)

There were also sizing difficulties associated with the patellofemoral brace, which sometimes led to the brace not fitting the participant as expected.

*There was one or two cases where the brace size wasn’t quite a match up for the guidelines on the box, so for the Bio‐skin, we needed to tweak for example if they measured up as a small, actually they might’ve needed a medium or a large.* (P02)

### 3.6 Brace Adherence Enhancing Strategies

#### 3.6.1 Brief MI

Despite many physiotherapists viewing brief MI as one of the most complex aspects of delivering the knee brace intervention (due to lack of experience), it was overall deemed acceptable, enjoyable and an important mechanism to encourage long‐term adherence to brace use.

*I think from the motivational interviewing point of view it’s an incredibly important part of getting patients to comply and that’s always the hardest part of any form of treatment is the compliance*. (P01)

Perceived barriers that impeded the use of brief MI included if the participant was not perceived as receptive, MI was not deemed appropriate because the participant was already *‘very well‐motivated’* to use the brace, and lack of time.

*if they are more direct and don’t kind of embrace some of that change or talk as much I don’t spend too long with it, I just kind of move on if you like because if they’re happy they’ve got a plan and if I’m happy they’ve got a plan I think that’s the main thing.* (P04)

Facilitators of using brief MI included previous experience, example phrases, the PROP OA case report form (used by physiotherapists to record intervention provision) acting as a prompt, the knee brace diary to support conversations and increasing confidence with practise.

*it’s just not something that I’m used to doing every day, but then the more you get into it the easier it is*. (P07)

#### 3.6.2 The Knee Brace Diary

Physiotherapists were generally positive about the use of the knee brace diary in the follow‐up appointment, reflecting that it helped to support discussions around brace adherence, increasing brace use, and facilitating use of brief MI techniques.

*The diary’s really useful for just starting that conversation (…) we can say so how could we get that or would you be interested in trying to do it more or do you want to have a chat about how we might want to think about ways of doing that, so it basically it helps with directing a conversation.* (P04)

Physiotherapists reflected that, for the most part, participants completed the diaries, brought them to their follow‐up appointment and found completing the diary helpful. However, some physiotherapists did question the usefulness of the diary (depending on the level of detail provided), wondering whether it was too burdensome for participants, and whether it would be completed accurately.

*I’m always sceptical about brace diaries, because they’ll just fill it in to tell you what they want*. (P06)

### 3.7 The Follow‐Up Appointment

Physiotherapists described how the follow‐up appointment was necessary and important to check the fitting of the brace, ensure that the participant could don and doff and walk in it correctly, monitor and address brace adherence, and check and address any side effects from wearing the brace (e.g. skin soreness).

*I think you have to [have the follow‐up appointment] cos there are so many things – well, not so many things, but things that can go wrong with the brace.* (P08)

### 3.8 Perceived Impact of the Knee Bracing Intervention

All physiotherapists reflected on experiences in which participants reacted positively to the brace after fitting, with some noting immediate easing of OA symptoms.

*Well they’re [patients] very pleased with it and some patient report and instantaneous feeling of satisfaction from having received the brace. So that’s always very nice to have.* (P06)

Physiotherapists felt that between the initial and follow‐up appointments, participants were adhering well to brace use.

*Patients have complied well, they’ve found it easy to take part in, they’ve been pleased with the results*. (P08)

However, some physiotherapists noted that participants at times had adverse reactions to the brace, which could be perceived as a consequence of being overly motivated and enthusiastic to feel the benefits of the brace.

*One patient who developed like a fungal infection from using the brace too much… when I gave the patient the brace they were so happy with the brace that they were, you know, so pleased to be part of the bracing element of the trial.* (P03)

Physiotherapists reflected that knee braces are not currently freely accessible by all patients with knee OA, and that evidence is needed to inform best practice. Offering the knee bracing intervention based on a clinical decision (i.e. to patients that will experience the most benefit) and giving patients the choice to accept or reject the bracing intervention after it was offered supported appropriate integration into NHS care.

*It’s entirely up to them. If they don’t want to have a brace you can’t force them to have a brace*. (P06)

## 4 Discussion

This study explored the experiences and perceived acceptability of a bracing intervention for physiotherapists participating in the internal pilot phase of the PROP OA RCT. The experiences of delivering the bracing intervention were generally positive and the bracing intervention was broadly perceived to be acceptable. Below, key findings are discussed, reflecting on the TFA domains, where appropriate.

The findings highlight that, overall, the training programme was broadly viewed positively by physiotherapists to support delivery of the complex bracing intervention *(global acceptability)*. Components of the training programme, including reading x‐rays, brace fitting, and brief MI, were novel but particularly valued by most physiotherapists, contributing to their *affective attitudes* towards the bracing intervention. Physiotherapists reflected on their *self‐efficacy* to deliver the intervention. Although some physiotherapists felt that the training programme had adequately equipped them with new skills, some physiotherapists lacked confidence in some areas (e.g., reading x‐rays and brief MI) and faced difficulty identifying protected time alongside their busy clinical commitments to engage fully with the training *(burden).* Physiotherapists described supplementing the PROP OA training with additional formal training, informal training (e.g., practising fitting braces on family and friends), and using the additional training materials provided (e.g., videos on brace fitting). Continual practise delivering the intervention was important to increase confidence by integrating multiple new skills (alongside delivering advice, information and exercise instruction and trial procedures) within a single treatment session. This confidence was perceived as important in facilitating adherence to the bracing intervention. This is plausible as increased confidence from healthcare professionals is likely to strengthen therapeutic alliances, which previous research has shown to positively impact on adherence and pain outcomes from non‐pharmacological treatments (Kinney et al. 2020; Moore et al. 2020).

The findings highlight that, overall, the bracing intervention was broadly viewed positively by physiotherapists *(global acceptability)*. Physiotherapists described feeling confident in delivering advice, information, and exercise instruction. They felt this reflected core elements of physiotherapy care, and their usual management of people with knee OA. Interestingly, one physiotherapist did acknowledge that it can be difficult to explain OA in a way that people can understand. The need to offer up‐to‐date and easy to understand explanations about OA is becoming increasingly recognised as important (Jinks et al. 2024). Offering advice in a way that is too complex or not person‐centred might mean that people are unable to use the information given to them. In addition, framing OA within a disease and impairment discourse (e.g., describing OA as a disease ‘wear and tear’ of cartilage, rather than ‘tear, flare and repair’ (Birrell and Johnson 2022)) could perpetuate the belief that ‘nothing can be done’ so as to reduce intervention uptake (Bunzli et al. 2021). The OA guidebook was recognised by some physiotherapists as helpful in delivering information and advice about OA. Continuing to offer this written information and focussing on optimising delivery of patient information and advice alongside intervention components that are less familiar to the physiotherapists are important to optimise the potential *(perceived) effectiveness* of the bracing intervention.

Experiences of providing and fitting knee braces were generally positive *(affective attitude)*, and the follow‐up appointment ‘made sense’ to check that the brace fit and for supporting brace adherence *(intervention coherence)*. Physiotherapists thought it was important that participants, where appropriate, were provided with the knee brace *(ethicality)*, because participants were perceived to be adhering to, and benefiting from, the brace *(perceived effectiveness)*. However, one challenging aspect of delivering the bracing intervention appeared to be determining the pattern of knee OA presentation to determine brace type. Whilst physiotherapists generally felt very confident in undertaking a knee examination, reading x‐rays and determining the predominant compartmental pattern based on clinical and x‐ray findings was sometimes felt to be difficult, particularly if clinical and x‐ray findings were discordant (*self‐efficacy*). Discordance between clinical and x‐ray findings in knee OA is well documented (Duncan et al. 2006) but there are no established, validated clinical classification rules for compartment‐specific knee OA. Instead, in keeping with clinical practice guidelines emphasising the importance of clinical assessment in knee OA diagnosis, physiotherapists were encouraged to give greater weight to their clinical assessment over radiographic presentation when these were discordant and were provided with guidance on patterns of signs and symptoms likely to be consistent with predominant patterns of knee OA based on previous research evidence (see Appendix 1 for further details).

Brief MI was another component of the bracing intervention that was challenging to deliver for some physiotherapists. Despite its complexity, it was overall deemed acceptable, enjoyable *(affective attitude)*, and an important mechanism through which to try to ensure the intervention was effective to support adherence to brace use *(intervention coherence)*. This is supported by previous research which has shown MI to be effective in increasing adherence to medication (Adler et al. 2017; Rathbone and Prescott 2017) and behavioural change (Borrelli et al. 2016; Lin et al. 2016) interventions. One physiotherapist perceived brief MI not to be relevant if the participant was ‘already motivated’, potentially demonstrating oversight of some key MI principles for example, reflective listening *(intervention coherence).*

### 4.1 Strengths and Limitations

Strengths of this study include its theoretical underpinning and robust methods (interviews being undertaken by a researcher not known to participating physiotherapists, and use of a multi‐disciplinary team to increase trustworthiness of analysis). Only nine physiotherapists were involved in the PROP OA internal pilot phase, limiting the sampling pool for this qualitative study. Furthermore, participating physiotherapists represent a cohort that are more experienced than the wider UK physiotherapy workforce. Participating physiotherapists were also more likely to have an interest in OA, bracing, and/or MI and were potentially more committed to continuing professional development. One physiotherapist declined (reason unknown), who might have had different views and experiences. These factors potentially limit the transferability of the findings (Johnson, Adkins, and Chauvin 2020). Interviews were completed 3 months after the RCT commenced recruitment, limiting our understanding of longer‐term intervention acceptability. Despite the small sample size, the data collected was rich, achieving high information power (Malterud, Siersma, and Guassora 2016). We acknowledge that, if delivered in the NHS as part of usual practice, other professional roles would also deliver bracing interventions. However, this was outside the scope of the PROP OA RCT.

### 4.2 In the Context of the Wider Literature

This qualitative study provides novel insights into the acceptability of physiotherapists delivering brace interventions for knee OA. We found that physiotherapists supplemented trial training with additional formal training, and experiential learning was important to increase confidence in delivering the multiple components of the bracing intervention. This was also found in a previous study in which physiotherapists were trained to deliver a complex intervention (a very low energy diet and exercise intervention for weight loss in people with knee OA) (Allison et al. n.d.). Our finding, that physiotherapists perceived advice, education, and exercise instruction to reflect core elements of physiotherapy care, and their usual management of people with knee OA mirrors the findings of several previous studies exploring physiotherapy management of people with knee OA (Hagen et al. 2016).

### 4.3 Research and Clinical Implications

The new knowledge generated in this study provides useful insights for the potential future implementation of the bracing intervention. This includes recognition that in addition to the training programme, experiential learning will be important in increasing physiotherapists' confidence in delivering the bracing intervention. In addition, some physiotherapists are likely to need ongoing support when integrating new skills within their clinical practice, for example, reading x‐rays, making decisions about brace allocation, and in use of brief MI.

## 5 Conclusion

Overall, the complex bracing intervention being tested within the PROP OA RCT was experienced positively by physiotherapists and was broadly perceived as acceptable. To support physiotherapists to deliver a complex bracing intervention, initial training should be supplemented with ongoing support and mentoring to optimise integration into existing clinical practice.

## Author Contributions

**Laurna Bullock**: data curation, formal analysis, writing–review and editing. **Melanie A. Holden**: conceptualization, methodology, data curation, supervision, formal analysis, funding acquisition, writing–original draft preparation, writing–review and editing. **Clare Jinks**: Conceptualization, methodology, data curation, supervision, formal analysis, funding acquisition, writing–review and editing. **Evans Atiah Asamane**: conceptualization, methodology, investigation, project administration, writing–review and editing. **Dan Herron**: conceptualization, investigation, writing–review and editing. **Belinda Borrelli**: conceptualization, funding acquisition, writing–review and editing. **Michael J. Callaghan**: conceptualization, funding acquisition, writing–review and editing. **Fraser Birrell**: conceptualization, funding acquisition, writing–review and editing. **Nicola Halliday**: data curation, project administration, writing–review and editing. **Michelle Marshall**: writing–review and editing. **Gail Sowden**: writing–review and editing. **Carol Ingram**; conceptualization, funding acquisition, writing–review and editing. **John McBeth**: funding acquisition, writing–review and editing. **Krysia Dziedzic**: Conceptualization, funding acquisition, writing–review and editing. **Nadine E. Foster**: conceptualization, funding acquisition, writing–review and editing. **Sue Jowett**: funding acquisition, writing–review and editing. **Sarah Lawton**: project administration, funding acquisition, writing–review and editing. **Christian D. Mallen**: conceptualization, writing–review and editing. **George Peat**: conceptualization, funding acquisition, methodology, supervision, writing–review and editing.

## Ethics Statement

Ethical approval to undertake the study was obtained from the Northwest ‐ Preston Health Research Authority Research Ethics Committee (IRAS Number: 247370; REC Reference: 19/NW/0183).

## Conflicts of Interest

CDM is director of the National Institute for Health and Care Research School for Primary Care Research (SPCR). All other authors declare no conflicts of interest.

## Data Availability

Data research is not found.

## Appendix A

**Appendix 1:** Guidance provided to support physiotherapists to determine, on clinical grounds, the predominant knee compartment affected (medial tibiofemoral, lateral tibiofemoral, patellofemoral, no clear predominant compartment) Table A1.

**TABLE A1:** Clinical features to consider when determining which compartment may be most affected.

	Think medial tibiofemoral joint if…	Think lateral tibiofemoral joint if…	Think patellofemoral joint if…
Previous surgery/injury	Previous total or partial medial meniscectomy^a^ ^,^ ^b^, medial meniscal repair^c^	Previous total or partial lateral meniscectomy, lateral meniscal repair	Previous patella subluxation and/or dislocation
Location of maximal pain/tenderness	Medial aspect of knee/medial joint line^d^	Lateral aspect of knee/lateral joint line^d^	Anterior aspect of knee/retropatellar^e^, ^f^, ^g^, ^h^, ^i^
Aggravating factors	Standing/walking^j^	Standing/walking^j^	Stair climbing, rising from sitting, kneeling, squatting^e^, ^f^, ^g^, ^h^, ^i^
Frontal malalignment	Varus^k^	Valgus	
Other tests	**Varus thrust during gait** ^l^ ^,^ ^m^	Valgus thrust during gait (rare)	Positive Clarke's test^k^
Effusion^g^ ^,^ ^k^			

^a^ Papalia et al. Br Med Bull 2011; 99:89–106. https://doi.org/10.1093/bmb/ldq043.

^b^ van Meer BL et al. Br J Sports Med 2015; 49:975–983. doi: 10.1136/bjsports‐2013–0932583.

^c^ Jones & Spindler. J Orthop Res. 2017; 35:1366–1374. doi: 10.1002/jor.23557.

^d^ Parsons et al. Ageing Clin Exp Res. 2018; 30:17–25. doi: 10.1007/s40520‐017–0847‐z.

^e^ Hinman & Crossley. Rheumatology (Oxford). 2007; 46:1057–62.

^f^ Crossley et al. BMC Musculoskelet Disord. 2008; 9:122. doi: 10.1186/1471–2474‐9–122.

^g^ Collins et al. Knee. 2017; 24:76–81.

^h^ Wyndow et al. J Foot Ankle Res. 2017; 10:19. doi: 10.1186/s13047‐017–0200‐y.

^i^ van Middelkoop et al. Semin Arthritis Rheum. 2018; 47:666–675. doi: 10.1016/j.semarthrit.2017.09.009.

^j^ Stefanik et al. Arthritis Care Res (Hoboken). 2018; 70:157–161. doi: 10.1002/acr.23238.

^k^ Peat et al. Arthritis Res Ther. 2012; 14:R63. doi: 10.1186/ar3779.

^l^ Chang et al. Arthritis Rheum. 2004; 50:3897–903.

^m^ Sharma et al. Arthritis Rheumatol. 2017; 69:2136–2143. doi: 10.1002/art.40224.

No single piece of information from the clinical assessment is likely to allow you to confidently determine which compartment of the knee is most severely affected by osteoarthritis. Instead, this is a judgement based on information on risk factors, pattern of symptoms, and findings on the physical examination. In the Clinical Eligibility Assessment, this judgement should be made without referring to patient X‐rays. Some of the features from the clinical assessment that would lead you to suspect predominant medial tibiofemoral joint involvement, predominant lateral tibiofemoral joint involvement (which is relatively uncommon), or predominant patellofemoral joint involvement are shown below.

Some additional points that you may find helpful:

- Lateral tibiofemoral joint OA is relatively uncommon. Medial tibiofemoral joint OA and patellofemoral joint OA are much more common.
- Varus malalignment and varus thrust during gait are very strong indicators of medial tibiofemoral joint osteoarthritis. When present, these findings should be weighted heavily in your judgement.
- There is some evidence that tibiofemoral joint osteoarthritis drives symptom and disease progression more than patellofemoral joint osteoarthritis. This suggests that when presented with a mixed picture of tibiofemoral and patellofemoral signs and symptoms, a bias towards tibiofemoral joint involvement may be justified.

## Appendix 2 Interview Topic Guide.

### PROP OA

#### Internal pilot

Physiotherapist Interview Topic Guide.

‐ Explain the interview study in line with the Physiotherapist Interview Information Leaflet.
‐ Audio‐record obtaining written informed consent

**Main Questions in bold,** *Prompts in italics.*

1. **Can you tell me a little bit about your professional background?**
2. **Can you tell me what training you have had to be able to deliver interventions as part of the PROP OA trial?**
  a. *Were any specific parts of the training particularly useful? Was there anything delivered in the training that you found was less useful or that could be improved?*
  b. *Would you have liked anything more in the way of either training or materials to use in practice?*
  c. *Do you think the training prepared you for your role?*
  d. *How confident did you feel in delivering your role after the training?*
3. **Are there any differences between what you do as part of PROP OA and what you would usually do?**

**Now I would like to hear about your experiences of deliv** **ering the PROP OA trial.**

4. **Can you outline what you would do in a typical PROP OA clinical eligibility assessment?**
  a. *How do you find completing this?*
  b. *Are there any challenges?*
5. **What is it like assessing and identifying the compartment of the knee affected by OA?**
  a. *How easy or difficult is this?*
  b. *Did anything help you to do this?*
  c. *Did you experience any challenges? Why?*
  d. *What would you like to help you with this?*
6. **How do you find reading the x‐rays?**
  a. *How easy or difficult was this?*
  b. *Did you experience any challenges?*
  c. *How did you overcome these?*
  i. *Did anything help you to read the x rays?*
7. **How do you find combining clinical examination and x‐ray results to make the decision about which type of knee brace to provide? Apart from these two, how do you find making the decision about which type of knee brace to provide?**
8. **How do you find delivering Best Primary Care as part of the trial within 20 min?**
  a. *How did you find delivering advice and exercises?*
  b. *How easy or difficult was this?*
  c. *Did you experience any challenges?*
  d. *How did you overcome these?*
  e. *Did anything help you?*
9. **Now I'd like to ask you how you are finding delivering the brace intervention?**
10. **How are you finding prescribing a brace to patients and supporting them to use it?**
  a. *How easy or difficult is this?*
  b. *Did anything help you to do this? How?*
  c. *Did anything make this more difficult for you to do?*
  d. *Did you experience any challenges?*
11. **What kinds of things are you focussing on in your follow‐up appointment?**
  a. *Adherence/is the type of brace being changed?*
  b. *Is the follow‐up appointment useful?*
12. **Has your decision about which type of knee brace to provide often changed? Why?**
13. **How do you find using the motivational interviewing techniques to encourage patient adherence?**
  a. *How easy or difficult was this?*
  b. *Did you experience any difficulties?*
  c. *Did anything help you to use the motivational techniques? How?*
14. **How do you find using the brace adherence diary to encourage patient adherence?**
  a. *How easy or difficult was this?*
15. **How have you found using the trial paperwork? (Prompts how did you find filling the paperwork around motivational interviewing) (the questions on the CRFs)**
16. **How confident did you feel in delivering the different components of PROP OA?**
  a. *Was there anything you were more confident with? Why?*
  b. *Was there anything you were less confident with? Why?*
17. **In general, how much effort did it take for you to deliver PROP OA?**
18. **Overall, is there anything in particular you found challenging when delivering the PROP OA interventions? Why?**
  a. *How could we help you with this?*
  b. *Did you experience any challenges putting PROP OA into practice at the physiotherapy service level?*
19. **Do you feel you have enough support to deliver the PROP OA trial?**
  a. *If so, can you describe this further?*
  b. If not, can you describe the support that would help you deliver the intervention?
  c. If things didn't go to plan or went wrong, how easy was it for you to get help/support from the study team?
20. **Have you discussed the approach used in PROP OA with your colleagues?**
  a. *How do you think your colleagues have found delivering PROP OA?*
21. **Might you continue to use elements of the intervention in the future?**
  a. *If yes, which ones? Why?*
  b. *How do you plan on taking forward delivering PROP?*
  c. *If no, why not?*
22. **How acceptable do you think PROP OA is to:**
  a. *BP patients?*
  b. *BP + B patients?*
23. **To what extent do you feel the interventions delivered in the trial have ethical implications for patient care? (Can you tell me more? In clinical practice, should patients be able to choose whether they receive a brace? In clinical practice, how fair do you think it is if some patients to receive a brace and others not to receive a brace?)**
24. **Is there anything else you would like to tell me today about being involved in and/or delivering the PROP OA interventions?**
  a. *Anything you wish to talk about that we haven't already covered?*

**Closing Statement:**

**On behalf of the PROP OA research team and Keele University, I would like to “Thank you” for participating in the PROP OA study and for taking the time to share with me your experiences of taking part.**
